# Thoraco-abdominal biomechanical model and dual-layer control method for soft robotic system with application to respiratory assistance

**DOI:** 10.3389/fbioe.2025.1581402

**Published:** 2025-04-25

**Authors:** Wenzhuo Zhi, Wei Zhao, Yan Zhang, Enming Shi, Yangfan Zhou, Bi Zhang

**Affiliations:** ^1^ State Key Laboratory of Robotics, Shenyang Institute of Automation, Chinese Academy of Sciences, Shenyang, China; ^2^ University of Chinese Academy of Sciences, Beijing, China; ^3^ Northeastern University, Shenyang, China; ^4^ Beihang University, Beijing, China

**Keywords:** human-robot interaction control, cross-domain biomechanics, wearable robots, duallayer control architecture, respiratory function enhancement

## Abstract

**Introduction:**

Respiratory dysfunction remains a critical challenge for patients transitioning from intensive care. However, existing assistive devices often fail to address both human-robot dynamic synchronization and patient safety due to the lack of interaction force control. Therefore, this study proposes a human-robot force interaction control strategy by integrating a flexible force sensor, aimed at achieving precise alignment between assistive forces and natural respiratory rhythms.

**Methods:**

In this study, a wearable respiratory assistive robotic system was developed to provide respiratory assistance by applying compressive force to the user’s abdomen through soft origami actuators. A thoracoabdominal biomechanical transmission analysis was conducted to reveal the cross-domain force transfer mechanisms. To improve the human-robot adaptability, a dual-layer control architecture for force-pressure coordinated control was designed. Besides, through hardware integration and system building, along with the implementation of interaction force control, the respiratory assistive robot achieves effective respiratory assistance control.

**Results:**

Within the 12–40 breaths/min effective respiratory rate range, PEF, MTV, and MV improved significantly. PEF had a 20.12% average increase, MTV a 19.69% average boost, and MV a 15.5% average rise. Statistically, PEF and MV improvements were highly significant across this range, while MTV was highly significant at 20 breaths/min. Respiratory MV measurements segmented by phase showed that the robot enhanced expiratory function and improved inspiratory function at certain rates within 12–40 breaths/min.

**Discussion:**

The proposed human-robot interaction control system integrates hardware and control systems. Tests on healthy subjects in the effective operating range show that the robotic system can enhance subjects' overall respiratory function and ventilation function, offering a technical reference for future respiratory-assist robot development.

## 1 Introduction

Intensive care unit-acquired weakness (ICU-AW) demonstrates a median incidence rate of 43% among critically ill patients (Vanhorebeek et al., 2020). Post-ICU discharge, these individuals continue to experience significant respiratory dysfunction (Herridge et al., 2016), particularly during transitional care phases from ICU to general wards or pre-discharge preparation (Fan et al., 2014). Despite liberation from invasive mechanical ventilation, there remains a persistent demand for respiratory assistance to enhance respiratory function and ensure safe interaction between the patient and the assistive device. Moreover, the large number of individuals with respiratory dysfunction, coupled with a shortage of hospitalization resources, has made home respiratory rehabilitation an increasing necessity. As a result, there is an urgent need for safe, lightweight, and portable respiratory assistance.

Current respiratory support devices mainly include manual assisted ventilation, positive-pressure mechanical ventilation, and negative-pressure mechanical ventilation. Manual assisted ventilation remains heavily dependent on healthcare provider intervention, resulting in significant resource allocation challenges (Goligher et al., 2016). While positive-pressure systems enable alveolar inflation through airway pressurization and are suitable for general ward use, their physiological mismatch with natural breathing mechanisms may lead to alveolar overdistension, increasing risks of barotrauma and adverse hemodynamic effects (Ding et al., 2018; Goligher et al., 2018). Negative-pressure ventilation systems, including biphasic cuirass ventilation with dual feedback mechanisms (Grasso et al., 2008) and continuous negative-pressure devices such as the Exovent and Breathe Global systems (Almeda et al., 2020), demonstrate superior physiological compatibility by mimicking natural respiratory mechanics. However, their substantial physical footprint necessitates prolonged bed confinement, paradoxically elevating risks of musculoskeletal deconditioning and pulmonary atelectasis. Despite advancements in respiratory support technologies, current clinical devices still face significant challenges in both biomechanical compatibility with natural breathing mechanisms and the provision of safe, portable respiratory assistance.

Recently, robotic technologies have been implemented for respiratory assistance. Zhu et al. developed rigid, multi-degree-of-freedom robotic systems to assist with thoracic expansion motions, using a fixed rhythm capable of mitigating respiratory workload (Zhu et al., 2015). To further improve human-robot synchronization during robotic respiratory assistance, Lee et al. designed an effort-synchronized respiratory assistance control strategy using a soft, wearable, cable-driven robotic system that optimally utilizes abdominal compression while aligning the assistance with the user’s spontaneous respiratory effort (Lee et al., 2015; Lee and Cho, 2017; Lee et al., 2022). Similarly, inspired by human respiratory physiology, Zhang et al. adopted pneumatic origami actuators to generate indirect pulmonary assistance through thoracoabdominal pressure transmission, while preserving biocompatibility via passive mechanical coupling, demonstrating their potential for precise and adaptable respiratory support (Zhang et al., 2022; Zhang et al., 2024a). However, existing robotic control strategies for respiratory assistance lack dynamic planning of interactive forces, resulting in poor human-robot adaptability.

A reliable respiratory sensor system is of great importance for the control of robotic respiratory assistance devices. Thus, this study focuses on the hardware aspects of respiratory sensors. Different sensor technologies possess unique technical features. Electrical impedance tomography (EIT) enables pulmonary ventilation visualization through dynamic impedance reconstruction (Tomicic and Cornejo, 2019), yet faces measurement accuracy degradation due to actuator-induced deformations that dynamically alter electrode-skin contact impedance. Inertial measurement units (IMUs) achieve respiratory phase tracking with millisecond-level latency and high sampling rates, though their dynamic precision is compromised by motion artifact-induced signal distortion (Liu et al., 2020). Ultrasound sensor techniques detect diaphragm displacement via Doppler shift analysis, demonstrating superior motion artifact resistance but suffering from probe positioning sensitivity that undermines measurement reliability (Shahshahani et al., 2018). In contrast, differential pressure-based airflow sensors combine stable signal output, sub-second response characteristics, and compact form factor, making them particularly suitable for real-time phase estimation in robotic respiratory assistance systems (Zhou et al., 2020).

Previously discussed sensor technologies play a crucial role in the human-robot interaction control system. However, when it comes to optimizing the assistive efficacy in respiratory human-robot interaction systems, the dynamic constraints of contact forces still pose ongoing challenges. Moreover, sudden changes in human-robot interaction forces are likely to cause localized stress concentration in the peritoneum. To tackle these issues, new approaches using flexible pressure sensor arrays (Oh et al., 2020; Zou et al., 2024) have emerged. These arrays allow actuators to adapt to anatomical dynamics, and by continuously monitoring the interaction between tissues and the device, they promote synergistic control between abdominal motion and robotic assistance.

Based on the preceding analysis, this study builds upon the design of pneumatic origami actuators. An interaction force feedback mechanism is implemented to constrain human-robot interaction forces within safety limits, ensuring patient comfort while maintaining therapeutic efficacy. By adopting a hybrid force-pressure control architecture based on pulmonary dynamics, the system achieves alignment between assistive forces and natural respiratory rhythms. Comparative trials demonstrated significant improvements in respiratory function, collectively validating the system’s capability to restore respiratory performance.

The contributions of this research are twofold:

(1) Integration of a soft single-phase respiratory assistance robot system: The hardware, including sensors, actuators, and a control unit, was constructed for precise parameter detection and effective assistance. Software integration involved developing control algorithms, an intuitive user interface, and communication protocols. Experiments demonstrated that this robot system effectively enhanced the overall respiratory function of healthy subjects.
(2) Development and application of a dual-layer force-pressure coordinated control strategy in the robot system: This strategy is designed to tackle the issue of the absence of dynamic force constraints on contact forces in current respiratory human-robot interaction systems. Through preliminary biomechanical analysis, a respiratory assistance strategy that more closely aligns with respiratory physiological characteristics is formulated.

## 2 Materials and methods

### 2.1 Robotic system integration and physiological support

**FIGURE 1:**
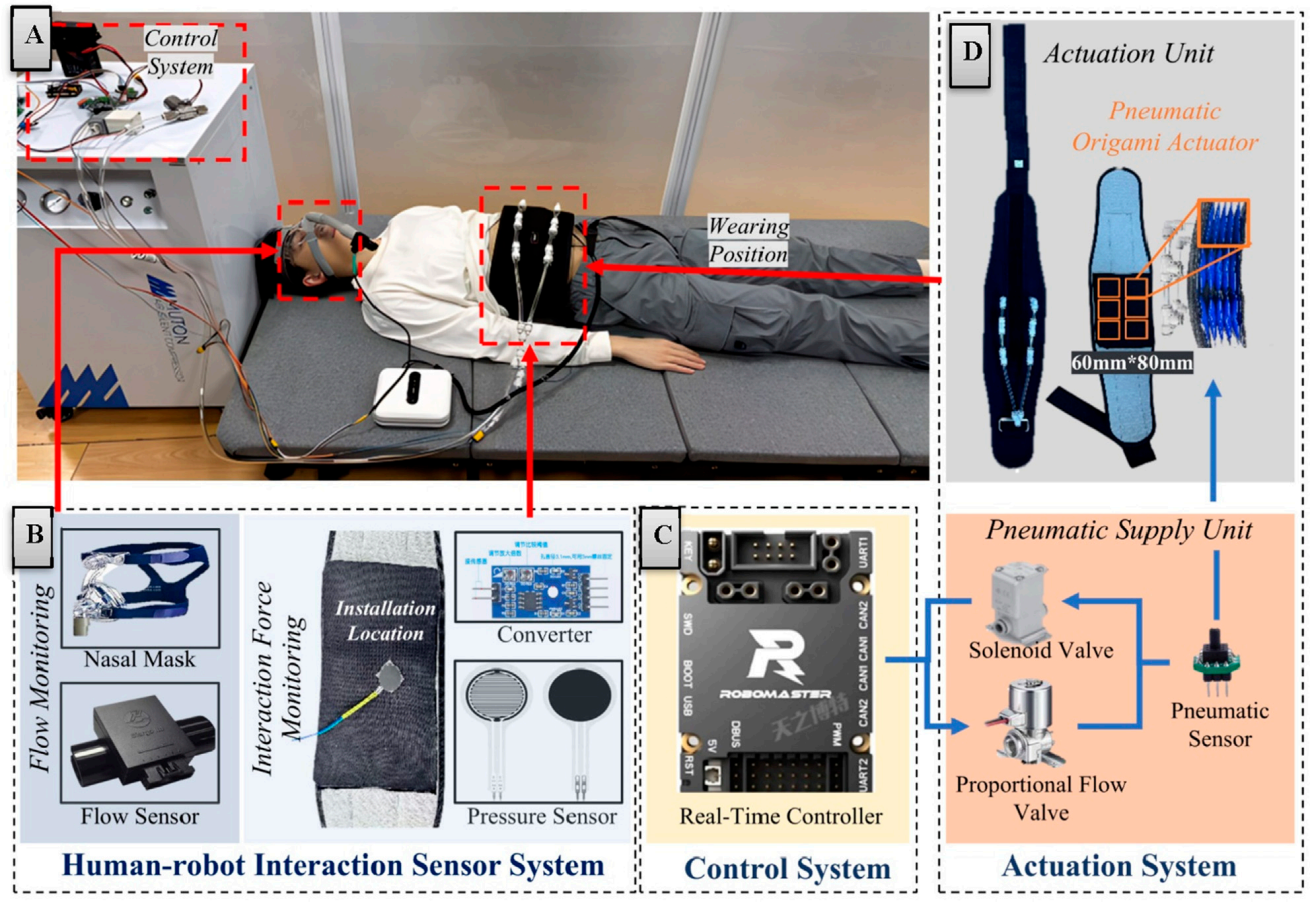
Hardware architecture and system integration diagram. **(A)** System integration diagram. **(B)** Human-robot interaction sensor system. **(C)** Control system. **(D)** Actuation system.

This chapter details the hardware architecture integration strategy of the respiratory assistive robotic system. The hardware framework consists of three core subsystems: an actuation system, a human-robot interaction sensor system and a control system, as systematically categorized. Through precision synchronization and interface optimization across subsystems, the platform ensures a highly integrated and reliable hardware foundation for subsequent operation and testing of human-robot interactive control systems. The comprehensive hardware configuration is illustrated in Figure 1A.

#### 2.1.1 Robotic system integration

The sensor system (Figure 1B) achieves detection of critical interactive parameters through real-time monitoring of respiratory flow and interaction forces. For respiratory parameter acquisition, a medical-grade respiratory flow sensor is employed to measure patients' breathing flow with high-precision accuracy. This sensor exhibits rapid dynamic response and high sensitivity, enabling precise detection of subtle respiratory rhythm variations to provide reliable physiological data for control strategies.

When it comes to the flexible force sensors integrated at the human-robot contact interface to obtain real-time interaction force data, several challenges are faced. The flexible nature of these sensors can cause measurement errors due to deformation during use, and motion artifacts can interfere with the accuracy of measurement.

To address these issues, sensor components with lower noise levels are selected to reduce interference at the source. The sensors are also attached to gaskets with relatively high rigidity to enhance stability and minimize errors from self-deformation. Additionally, the sensors are installed in the parts of the actuator that experience relatively slight deformation to avoid motion artifact interference.

Furthermore, in the design of the control system, the reliance on the contact force signals from flexible pressure sensors is lessened. The interaction force feedback is predominantly used as a signal for planning the desired contact force. This approach enables the system to generate assistive forces that can adapt to the human body more efficiently, even when there are some errors in the sensor data. In this way, the overall stability and reliability of the human-robot interaction control system are ensured.

In signal processing, a hardware-digital hybrid filtering strategy effectively suppresses high-frequency electromagnetic interference and environmental noise, significantly enhancing signal quality. The coordinated operation of these sensor mechanisms ensures system reliability and safety across diverse operational scenarios. The entire sensor system interfaces with the control system through standardized communication protocols, operating at a 200 Hz sampling frequency to meet real-time human-robot interaction requirements.

Building upon the sensor system, the control system architecture employs a hierarchical design combining an industrial PC and real-time controller to achieve dynamic adjustment and real-time response of robotic assistive forces. The industrial PC (Intel NUC, i5 processor) primarily operates the human-robot interface and upper-level control scripts, while the real-time controller (STM32F407, 168 MHz) communicates with the upper computer via CAN bus (1 Mbps) (Figure 1C). Functioning as the computational core, the real-time controller executes high-frequency sampling (200 Hz) of human-robot interaction signals, assistive force generation (100 Hz), and pressure tracking (100 Hz). System communication delay is strictly constrained within 5 ms to ensure real-time performance and motion control precision.

The electrical system utilizes a DJI portable smart power supply (nominal 22.8 V, 5,000 mAh), with voltage conversion through step-down DC-DC modules. Digital components including the controller (STM32F407), sensor interfaces, and communication circuits operate at 3.3 V/5 V, while pneumatic elements (proportional flow valves, solenoid valves) directly utilize the raw voltage. All functional modules incorporate essential power filtering circuits, with analog signals transmitted differentially. Signal conditioning circuits normalize sensor outputs to standard voltage ranges (0–3.3 V). The system features 12-bit ADC resolution alongside overcurrent and overvoltage protection mechanisms to enhance operational safety and reliability.

Complementing the electrical and control systems, the robotic actuation system (Figure 1D) comprises a pneumatic supply unit and an actuation assembly. The pneumatic supply unit integrates an oil-free silent air pump (rated flow: 20 L/min, operational noise <45 dB) with flow control components to meet medical rehabilitation noise requirements. The flow acquisition subsystem employs a high-precision proportional flow valve with sensitivity below 2% F.S. and repeatability within ±3% F.S., enabling continuous airflow modulation.

The actuation assembly adopts a soft wearable design consisting of a belt component and pneumatic compression modules. The belt features breathable mesh material with bidirectional Velcro fasteners (waist circumference: 850–1,200 mm) and anti-slip structures. Six origami-inspired pneumatic chambers (60 mm × 80 mm) provide directional compression through high folding-expansion ratios (Zhang et al., 2024a). The system limits maximum output force to 400 N through coordinated control of silent pumps, proportional valves, and solenoid valves. Safety mechanisms include pressure limiting and emergency venting functions. The wearable subsystem weighs <5 kg, ensuring ergonomic comfort during prolonged use. The origami structure is chosen as the basic driving unit for several reasons. Its unique folding and unfolding features allow for significant deformation and a high folding-expansion ratio. This enables the designed pneumatic chambers to generate a large compressive force upon inflation and be easily folded for storage when not in use, thus enhancing the device’s practicality and wearability. Moreover, the origami structure exhibits relatively stable mechanical properties. It can maintain consistent mechanical characteristics during multiple usage cycles, which is essential for ensuring the system’s reliability and stability.

#### 2.1.2 Physiological principle of robotic respiratory assistance

The selection of the actuator’s working site is of utmost importance for the proper functioning of a respiratory assistance robotic system. This section meticulously analyzes the reasons for choosing this specific site.

**FIGURE 2:**
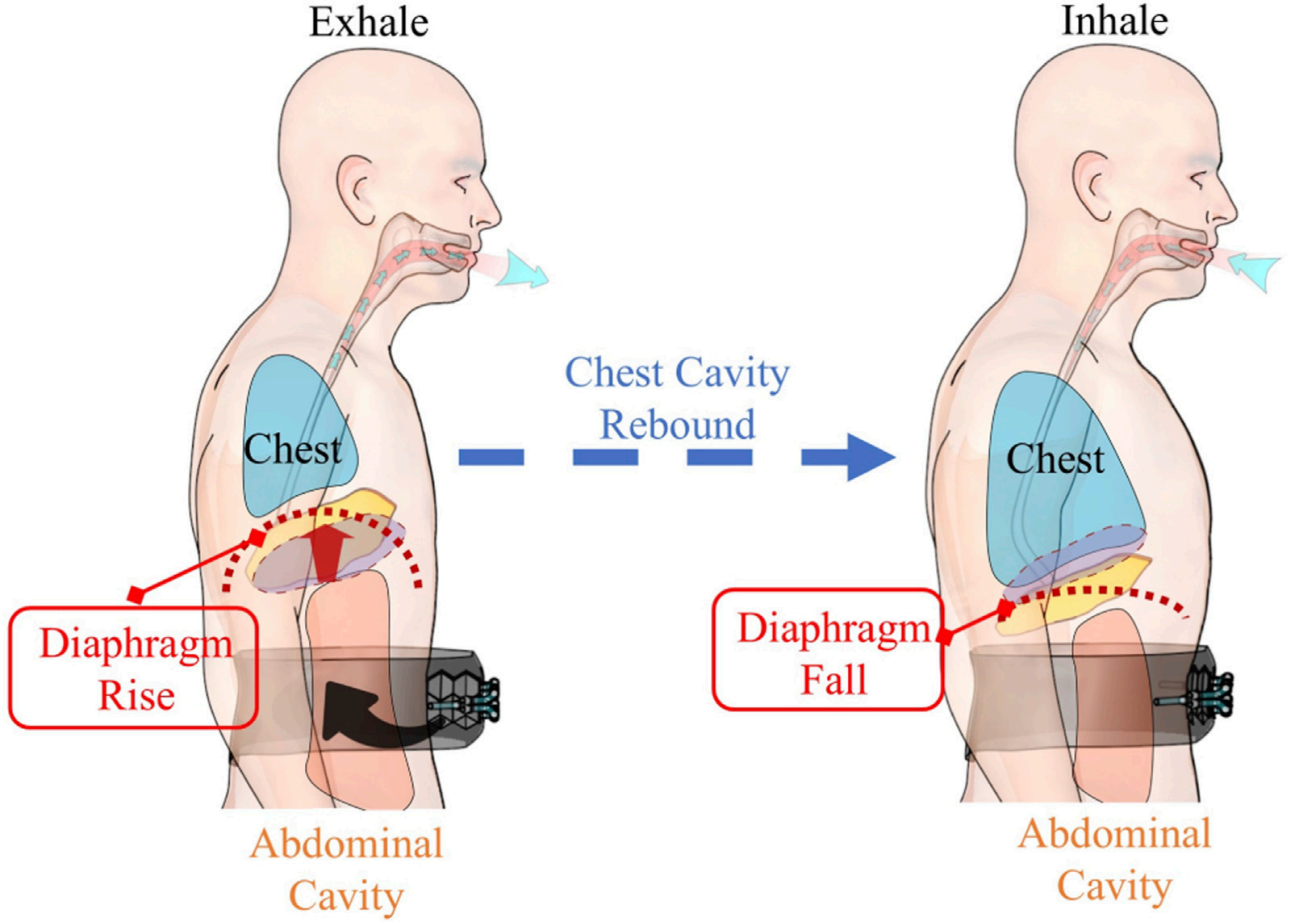
Mechanism of abdominal assistance in respiratory phases.

Human respiratory activities mainly rely on the coordinated efforts of respiratory muscles. The diaphragm (Figure 2), as the principal respiratory muscle, accounts for ∼70%–80% of the total respiratory capacity (Kokatnur and Rudrappa, 2018). A typical respiratory process comprises an active inspiratory phase and a passive expiratory phase (Hamasaki, 2020). During exhalation, air is expelled mainly due to the elastic recoil of the thoracic cavity and the contraction of abdominal wall muscles (Hamasaki, 2020). Clinical studies (Kim and Lee, 2013) have revealed that the contraction of abdominal wall muscles can effectively increase intra-thoracic pressure, thus facilitating the expiratory process.

Based on the above-mentioned physiological mechanisms, our system applies auxiliary force to the abdominal region during the expiratory phase (Figure 2), simulating and enhancing the natural contraction of the abdominal wall muscles. This design presents two significant advantages. Firstly, by augmenting the contraction force of the abdominal wall muscles during exhalation, it can remarkably improve expiratory efficiency (Kim and Lee, 2013). Secondly, as the system does not interfere with the active inspiratory phase, it can better adapt to the patient’s natural breathing patterns (Hamasaki, 2020), enhancing human-robot compatibility.

Notably, during the subsequent inspiration, the thoracic cavity, which was compressed during exhalation, rebounds. This rebound generates a negative pressure within the thoracic cavity, which plays a vital role in drawing air into the lungs, in line with the body’s normal respiratory processes. This physiological analysis not only validates the rationality of the actuator working site selection but also provides a solid theoretical basis for optimizing the system’s modeling and control strategies.

### 2.2 Dynamics transmission mechanism of robotic-assisted force in thoracic-abdominal cavities

**FIGURE 3:**
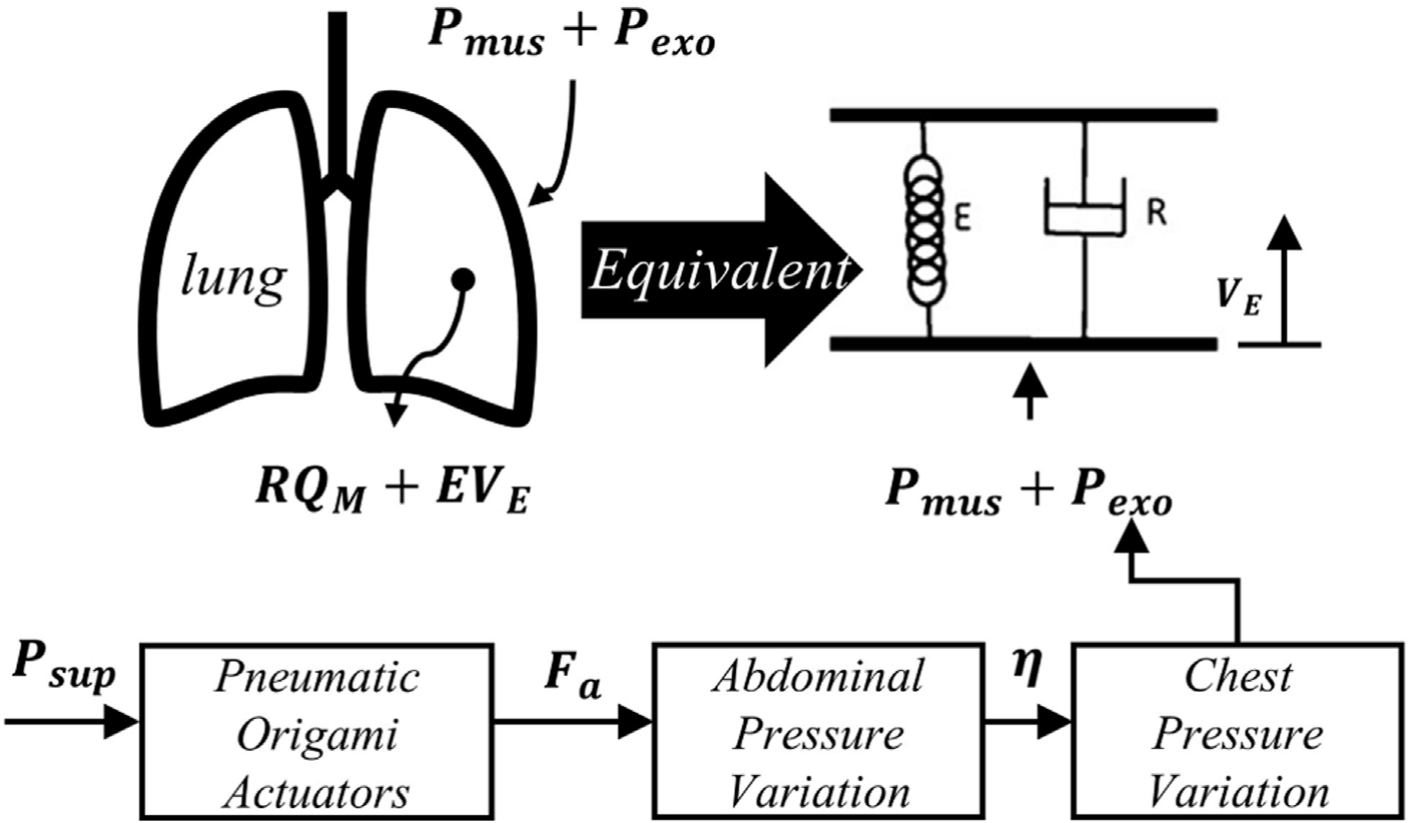
Schematic diagram of the equivalent model of lung dynamics and pressure transmission in respiratory assistance.

To investigate the cross-domain mechanical interactions of pneumatic compression components, this chapter establishes a mechanical transmission analysis framework from the actuation unit to physiological responses (Figure 3). This framework decouples the respiratory assistance process into three core aspects: the force-pressure characteristics of pneumatic compression components, the mechanical transmission mechanism in the thoracoabdominal cavity, and the dynamic responses of the respiratory system. This systematic analytical approach reveals the cross-domain mechanical interaction mechanisms among these subsystems during respiratory assistance, providing theoretical foundations and design references for subsequent control strategies.

#### 2.2.1 Mechanical characterization of pneumatic compression components

**FIGURE 4:**
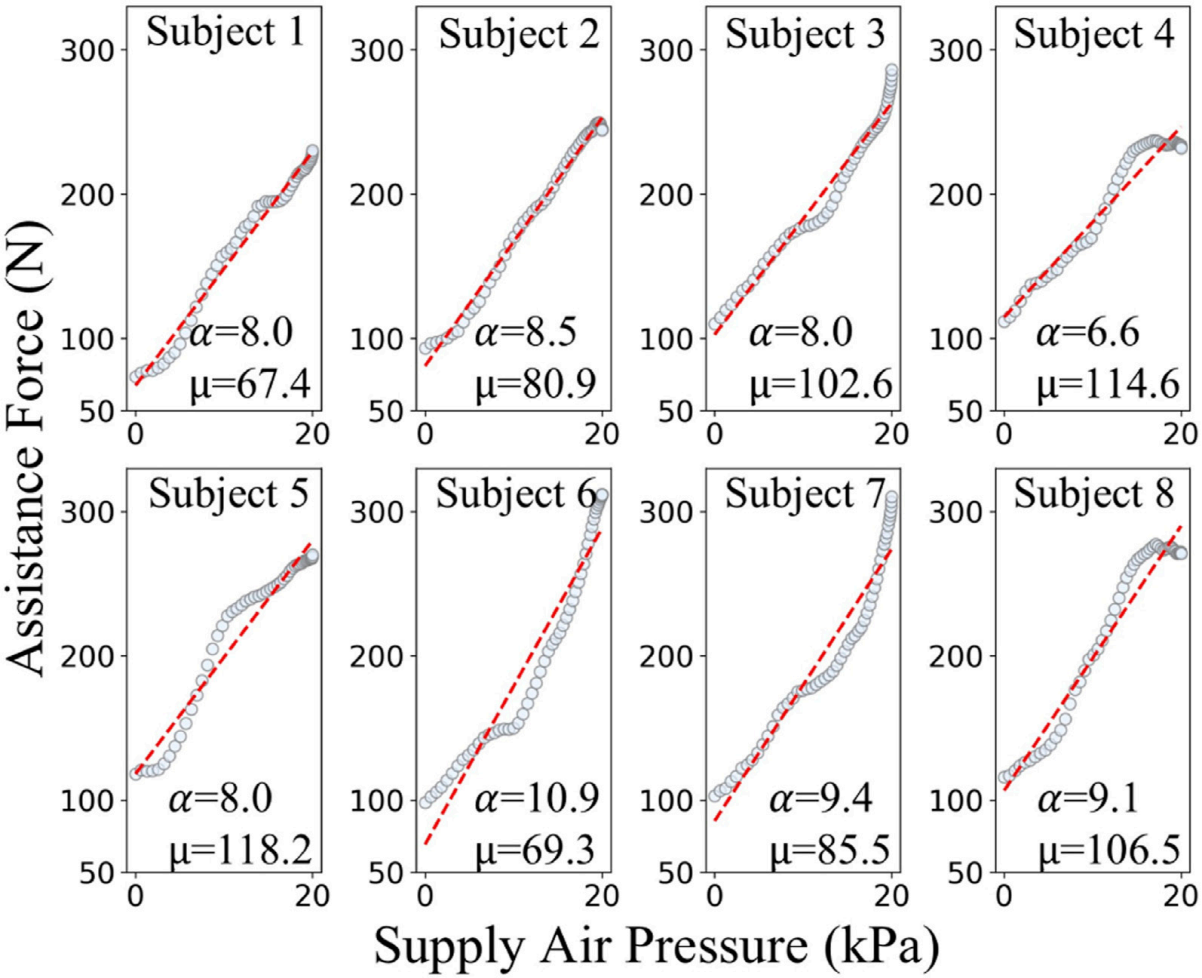
Experimental and fitted relationships between supply air pressure and assistive force of pneumatic origami actuators for different subjects.

To analyze the mechanical properties of the pneumatic compression components, this study employs a data-driven approach for mechanical characterization. Eight healthy subjects (age: 25 ± 3 years) were recruited for data acquisition. Among these eight subjects, the data points in Figure 4 specifically stem from eight different individuals (S1–S8). Due to the variance in mechanical properties like elasticity and damping of the human abdomen among individuals, the relationship between the supply pressure of the actuator acting on the abdomen and the generated assistive force varies remarkably from one subject to another. Participants wore the pneumatic compression components to complete a standardized 60-s breathing test. During this test, the system synchronously recorded the supply pressure $P_{\sup}$ and assistive force $F_{a}$ . As depicted in Figure 4, for subjects S1–S8, the experimentally derived force-pressure characteristic curve shows a strong linear correlation, with a Pearson correlation coefficient mean of 0.9885. This indicates a highly significant linear relationship between the supply pressure and the assistive force generated by the pneumatic compression components.

Experimental results demonstrate that within the system’s rated operating range (supply pressure: 0–20 kPa), the generated assistive force ranges from 0 to 400 N. To ensure safety, in this study, limits are imposed on both the supply pressure and the assistive force. Specifically, the supply pressure is limited to 20 kPa and the assistive force threshold is set at 400 N. Analysis of the respiratory data from subjects S1–S8 established a linear mechanical relationship for the pneumatic compression components during inflation, as shown in Equation 1:

$$F_{a}=\alpha \cdot P_{\sup}+\mu \;\left(0\leq P_{\sup}\leq 20\;kPa\right)$$ (1)

where α , μ denote the force transmission gain coefficients, offers three advantages: it bypasses complex mechanical modeling, simplifies implementation, and achieves high computational efficiency. By calculating the parameters for each subject, we obtained the results presented in Table 1.

**TABLE 1:** Calibration of key parameters in the force transmission model for different subjects.

Subject ID	α	μ	*R* ^2^	Pearson r
S1	8.09	67.41	0.982	0.991
S2	8.59	80.92	0.992	0.996
S3	8.01	102.64	0.969	0.984
S4	6.60	114.68	0.951	0.975
S5	8.08	118.23	0.943	0.971
S6	10.95	69.30	0.928	0.963
S7	9.41	85.57	0.927	0.962
S8	9.17	106.59	0.952	0.975

As shown in the Table 1, the model exhibits excellent fitting performance across the operational range, with all the coefficients of determination (*R* ^2^ > 0.927) and Pearson correlation coefficients (*r* > 0.962). These results effectively characterize the mechanical behavior of the pneumatic compression components.

#### 2.2.2 Biomechanical transfer mechanism in thoracoabdominal cavities

To establish a quantitative mapping relationship between robotic-assisted force $F_{a}$ and the resultant pulmonary pressure $P_{exo}$ , a biomechanics-based force-pressure transmission model was developed. The model simplifies the system with four assumptions: (1) the abdominal cavity was abstracted as a closed fluid-filled chamber obeying static pressure equilibrium (Pascal’s law); (2) the diaphragm was modeled as a quasi-static elastic membrane with linear deformation governed by equivalent stiffness; (3) the thoracic cavity was treated as a rigid chamber whose volume changes were solely driven by diaphragmatic displacement; (4) respiratory muscles were assumed to remain relaxed.

Based on these assumptions, the force-pressure transmission was decomposed into four cross-domain coupling stages. First, the robotic assistive force $F_{a}$ was converted to abdominal pressure $P_{ab}$ via static pressure transmission:

$$P_{ab}=\frac{F_{a}}{A}$$ (2)

Where A is the equivalent pressure-transmission area of the contact region. Second, abdominal pressure variation $\Delta P_{ab}$ induced diaphragmatic deformation, leading to abdominal volume change $\Delta V_{ab}$ :

$$\Delta V_{ab}=C_{ab}\cdot \Delta P_{ab}$$ (3)

Where $C_{ab}$ is the diaphragmatic compliance coefficient. Third, the abdominal volume change $\Delta V_{ab}$ altered thoracic volume, generating thoracic pressure variation $\Delta P_{th}$ based on thoracic elasticity (elastic coefficient $k_{th}$ ) and initial thoracic volume $V_{0}$ :

$$\Delta P_{th}=\frac{-k_{th}\cdot \Delta V_{ab}}{V_{0}}$$ (4)

The thoracic pressure variation $\Delta P_{th}$ acts on the lungs through tissue pressure transmission efficiency η and the transmission ratio η is subject-specific. Finally, the effective pressure increase in the lungs due to the actuation of robot ${\;P}_{exo}$ can be expressed as:

$$P_{exo}=\Delta P_{th}\cdot \eta$$ (5)

Combining Equations 2–5 by eliminating intermediate variables yields the quantitative relationship between $P_{exo}$ and $F_{a}$ :

$$P_{exo}=-\left(\frac{k_{th}\cdot C_{ab}\cdot \eta }{A\cdot V_{0}}\right)\cdot F_{a}$$ (6)

According to Equation 6, letting the transmission coefficient $\delta =\frac{k_{th}\cdot C_{ab}\cdot \eta }{A\cdot V_{0}}$ , the model simplifies to a linear expression, as shown in Equation 7:

$$P_{exo}=\delta \cdot F_{a}$$ (7)

Where δ represents the force-pressure transmission efficiency from robotic assistive force to pulmonary pressure through thoracoabdominal tissues.

#### 2.2.3 Theoretical analysis of pulmonary dynamics

To characterize the pulmonary effects of $P_{exo}$ and $P_{mus}$ , a simplified first-order spring-damper lung dynamics model (Saatci and Saatci, 2020) comprising airway resistance R and lung elastance E was adopted (Figure 3). In this paper, it is applied to generate the assistive force curves in 2.3.1, enabling simulation and analysis of the interaction between the robotic assistance and human respiratory system. The total pulmonary pressure balance equation is expressed as:

$$R\frac{{dV}_{lung}}{dt}+EV_{lung}+P_{mus}+P_{exo}+P_{PEEP}=0$$ (8)

Where: $V_{lung}$ represents the lung air volume, and $V_{lung}^{0}$ denotes the residual volume. $P_{exo}$ is the effective pressure increase in the lungs due to the actuation of the robot. $P_{mus}$ is the pressure in the lungs generated by human respiratory muscles. Focusing on respiratory motion dynamics rather than static lung volume, Equation 8 was reformulated as Equation 9:

$$RQ_{M}+EV_{E}=P_{mus}+P_{exo}$$ (9)

With substitutions $V_{lung}=-V_{T}+V_{lung}^{0}$ and ${\mathrm{EV}}_{lung}^{0}+P_{PEEP}=0$ , where $Q_{M}$ (Mean Airflow Velocity) and $V_{E}$ (Minute Ventilation) correspond to experimentally measured respiratory flow and volume. A state-difference identification scheme was proposed to address the unmeasurable $P_{mus}$ . Subjects maintained metronome-guided breathing rhythms during data acquisition, with system responses recorded under robotic assistance (Group A) and natural breathing (Group B). The parameter estimation equation via the least squares method was formulated as:

$${\left[R\;E\right]}^{T}={\left(H^{T}H\right)}^{-1}H^{T}y$$ (10)

According to Equation 10, where the measurement matrix H and output vector y integrate sampled $Q_{M}$ , $V_{E}$ , and $F_{a\;}$ data at 200 Hz. Matrix components are defined as:

$$H=\left[\begin{array}{ccc} Q_{M}^{A}\left(1\right)-Q_{M}^{B}\left(1\right) & \ldots & Q_{M}^{A}\left(N\right)-Q_{M}^{B}\left(N\right)\\ V_{E}^{A}\left(1\right)-V_{E}^{B}\left(1\right) & \ldots & V_{E}^{A}\left(N\right)-V_{E}^{B}\left(N\right) \end{array}\right]$$

$$y={\left[\begin{array}{ccc} \delta \cdot F_{a\;}\left(1\right) & \ldots & \delta \cdot F_{a\;}\left(N_{1}\right) \end{array}\right]}^{T}$$

The identified parameters R = 2.7 cm H_2_O⋅s/L and E = 4.8 cm H_2_O/L fall within the reported physiological range for healthy adults.

### 2.3 Design and implementation of dual-layer control architecture for force-pressure coordinated control

A dual-layer control architecture for force-pressure coordinated control was proposed, designed based on respiratory dynamics to generate assistive forces synchronized with human breathing rhythms. The architecture comprises three main modules: (1) The High-Level Controller designs the desired pressure profile based on pulmonary dynamics and respiratory flow amplitude, generating physiologically consistent assistive forces that are matched with the physiological characteristics of human respiration. (2) The Low-Level Controller takes $F_{a}^{ref}$ output by the High-Level Controller as a reference. It dynamically plans $F_{a}^{ref}$ based on the real-time feedback of $F_{\mathrm{int}}$ to generate $F_{plan}$ . Subsequently, $F_{plan}$ is converted into the corresponding $P_{\sup}^{ref}$ through the established force-pressure mapping relationship. Finally, it uses a Proportional-Derivative (PD) control algorithm to precisely track $P_{\sup}^{ref}$ . This ensures that the actual pressure supplied by the actuator closely follows the desired value, thus accurately implementing the assistive force plan and guaranteeing the high-precision execution of the respiratory assistance task.

**FIGURE 5:**
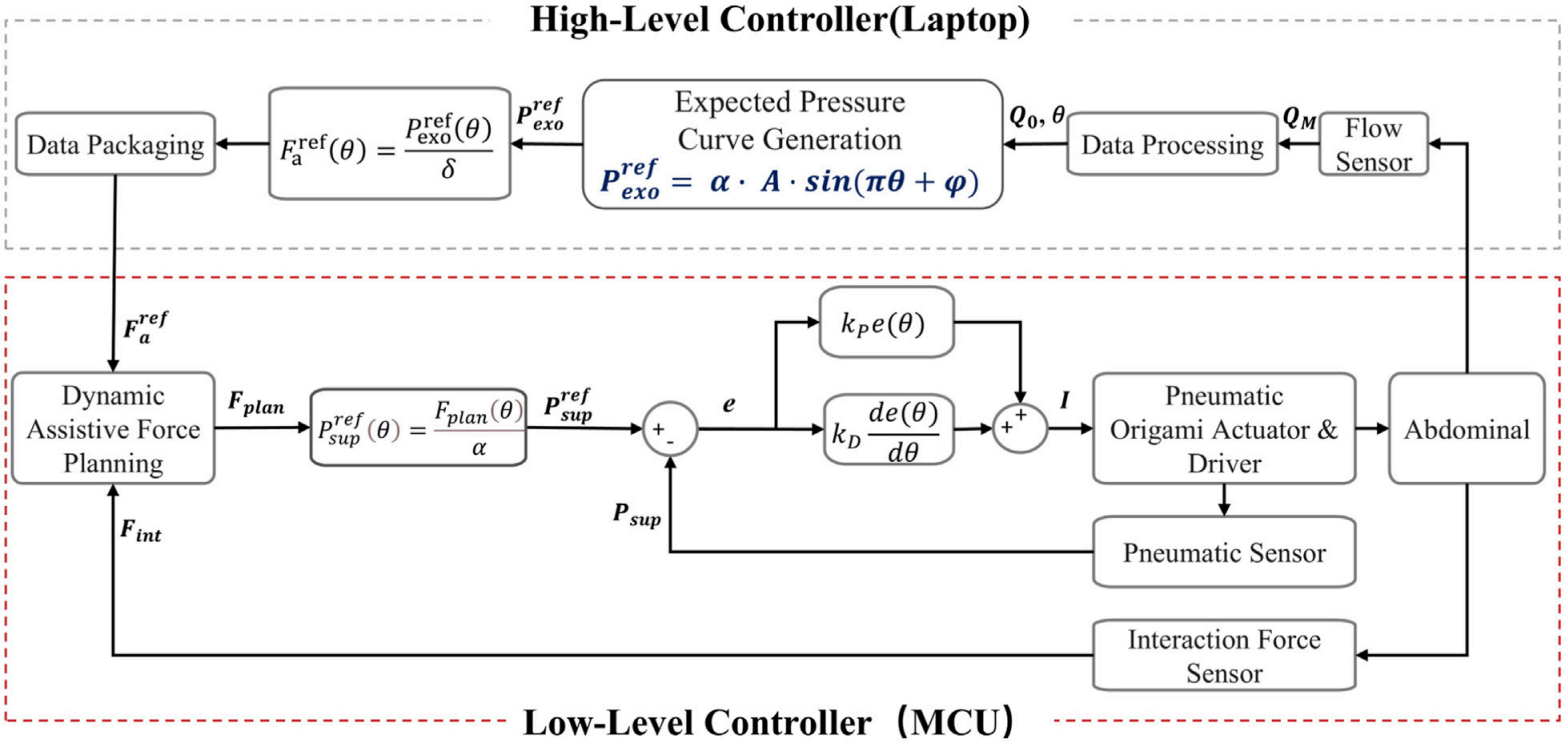
Schematic diagram of the dual-layer control architecture for force-pressure coordinated control.

The control system establishes a force-pressure mapping relationship and implements a two-layer control strategy. In the High-Level Controller, an assistive force generation mechanism based on respiratory dynamics is implemented. In the Low-Level Controller, dynamic assistive force planning and pressure tracking are carried out. This ensures that the actuator output precisely matches the respiratory demands. This control architecture not only enhances the coordination of human-robot interaction but also improves the safety and adaptability of respiratory assistance for patients. Figure 5 summarizes the control framework of this chapter.

#### 2.3.1 Assistive force design based on flow amplitude and pulmonary dynamics

In the expiratory phase assistance control, a specific method is employed to determine the key parameter θ . Binary phase segmentation is first completed through specific processing. Subsequently, based on the characteristics of the respiratory phase, the continuous expiratory phase θ is calculated as $\theta =\frac{V_{ex}^{k}}{V_{ex}^{k-1}}$ , where $V_{ex}^{k}$ is predicted from $V_{ex}^{k-1}$ .

Due to the structural limitations of the actuation system, this study focuses on abdominal compression assistance during the expiratory phase. Consequently, the control system implements closed-loop control of assistive forces only during expiration, where the expiratory phase $\theta \in \left[0,1\right]$ characterizes the current expiratory progress. The core objective of respiratory assistance robots is to enhance respiratory function (such as minute ventilation $Q_{M}$ and expiratory volume $V_{E}$ ) while reducing the respiratory load. Considering this, the ideal control strategy should be centered around the pressure $P_{mus}$ generated by the respiratory muscles.

However, direct measurement of $P_{mus}$ is technically challenging and lacks reliability, making it unsuitable as a feedback signal for real-time control. Therefore, this study proposes indirect control of pulmonary pressure by designing an assistive force curve synchronized with the respiratory rhythm. Although the reference assistive pressure curve $P_{exo}^{ref}$ for natural breathing cannot be directly obtained, it can be reconstructed indirectly using the easily measurable respiratory flow Q . Based on experimental observations, the flow during natural breathing exhibits a near-trigonometric variation, which can be expressed as shown in Equation 11:

$$Q\left(\theta \right)=Q_{0}\cdot \sin\left(\pi \theta \right),\theta \in \left[0,1\right]$$ (11)

Where $Q_{0}$ is the flow amplitude. Building on the pulmonary dynamics theory (Equation 9), the respiratory system was simplified as a first-order spring-damper system comprising alveoli, airways, and the thoracic cavity. Its key parameters, airway resistance R and lung elastance E , were obtained through offline identification. Within this model framework, the pressure $P_{mus}$ generated by respiratory muscles must overcome two primary resistances, the pressure drop due to airway resistance R can be described as presented in Equation 12:

$$P_{R}\left(\theta \right)=R\cdot Q\left(\theta \right)$$ (12)

And the alveolar elastic pressure (related to volume change):

$$P_{E}\left(\theta \right)=E\cdot V\left(\theta \right)$$ (13)

Where E is the lung elastance and $V\left(\theta \right)$ is the volume change. By integrating $\left(\theta \right)$ , the volume change $V\left(\theta \right)$ is derived as:

$$V\left(\theta \right)=\int Q\left(\theta \right)d\theta =-\left(\frac{Q_{0}}{\pi }\right)\cos\left(\pi \theta \right)+C$$ (14)

Given the periodic nature of respiratory motion, an appropriate reference point can be chosen such that C = 0. Combining Equations 13–15, the expression for $P_{mus}$ is obtained:

$$P_{mus}\left(\theta \right)=P_{R}\left(\theta \right)+P_{E}\left(\theta \right)=A\cdot \sin\left(\pi \theta +\varphi \right)$$ (15)

Where the pressure amplitude $A=Q_{0}\sqrt{R^{2}+{\left(\frac{E}{\pi }\right)}^{2}\;}$ (unit: cmH_2_O) represents the maximum muscle pressure required to generate a flow amplitude $Q_{0}$ , and the phase difference $\varphi =\arctan\left(-\frac{E}{\pi R}\right)$ reflects the lead of the muscle pressure waveform relative to the flow waveform. This lead arises because changes in pulmonary pressure are a prerequisite for flow generation. Based on this theoretical analysis, the expression for robotic assistive pressure is designed to achieve synchronization with the human respiratory rhythm, as shown in Equation 16:

$$P_{exo}^{ref}\left(\theta \right)=\alpha \cdot A\cdot \sin\left(\pi \theta +\varphi \right)$$ (16)

Here, α represents the pressure gain parameter. To ensure the safety of the assistance process, this study adheres to the guidelines of the American Thoracic Society (ATS) and related clinical practice recommendations, setting the safety threshold for total airway pressure at 30 cmH_2_O, as shown in Equation 17:

$$P_{mus}\left(\theta \right)+P_{exo}\left(\theta \right)\leq 30\;cmH_{2}O$$ (17)

The configuration of the pressure amplitude A and the gain parameter α must strictly comply with this safety constraint. This control strategy, based on the pulmonary dynamics model, provides a theoretical foundation for achieving synchronization and safety between assistive forces and respiratory motion.

It should be emphasized that in this study, we utilize the parameters R and E from the pulmonary dynamics model (Equation 8), which is a classic model validated in clinical settings, to determine the rated value of the assistive force. This model has been well established and verified in clinical practice, providing a reliable theoretical foundation for our research.

The values of these parameters, similar to gain parameters, depend on offline respiratory identification and adjustments during actual use. Based on clinical recommendations and practical experience, we have provided the adjustment ranges of these parameters in Table 2. Although the control system does not overly rely on the absolute accuracy of the model, the main purpose of incorporating this model is to offer theoretical guidance for the control strategy, ensuring its effectiveness and applicability in real-world scenarios.

**TABLE 2:** Parameter range of human-robot interaction control system.

Type	Parameter	Unit	Value
Thoracoabdominal mechanical transfer model	α	N/kPa	5–15
	μ	N/kPa	50–110
	δ	kPa/N	0.1–0.5
Pulmonary dynamics	R	cmH_2_O⋅s/L	1.5–5
	E	cmH_2_O/L	2 to 10
Control system	β	—	0–1
	B	—	0.1–10.0
	K	—	0.1–5.0
	$k_{P}$	—	1.0–10.0
	$k_{D}$	—	0.1–1.0

#### 2.3.2 Dual-layer control strategy for force-pressure coordinated control

Based on the analysis of the trans-thoracic-abdominal assistive force transmission mechanism in Chapter 2.2.2, a dual-layer control architecture for force-pressure coordinated control was proposed, aiming to enhance human-robot synchronization, safety. The architecture leverages two key mapping relationships established in Chapter 2.2.2: the force-pressure characteristics of the pneumatic compression component and the thoracoabdominal biomechanical transmission mechanism, where α is the force-pressure conversion coefficient and δ is the mechanical transmission coefficient. These models collectively form the theoretical foundation for control quantity conversion. Equation 7 transforms the desired pulmonary pressure $P_{exo}^{ref}$ into the desired assistive force $F_{a}^{ref}$ , while Equation 1 maps the planned force $F_{plan}$ to the actuator’s desired pressure $P_{\sup}^{ref}$ .

Based on these mapping relationships, the control strategy was implemented as follows. First, given the respiratory assistive pressure curve $P_{exo}^{ref}$ , the desired assistive force was derived as shown in Equation 18:

$$F_{a}^{ref}\left(\theta \right)=\frac{P_{exo}^{ref}\left(\theta \right)}{\delta }$$ (18)

The control objective of the assistive force dynamic planning is to generate the planned force $F_{plan}$ through a dynamic force planning control law based on the feedback of the interaction force $F_{\mathrm{int}}$ , as depicted in Equation 19:

$$F_{plan}\left(\theta \right)=F_{a}^{ref}\left(\theta \right)-B\cdot \frac{dF_{\mathrm{int}}\left(\theta \right)}{d\theta }-K\cdot F_{\mathrm{int}}\left(\theta \right)$$ (19)

Subject to: $0\leq F_{a}^{ref}\left(\theta \right)\leq F_{\max}$ , where the damping term $B\cdot \frac{dF_{\mathrm{int}}\left(\theta \right)}{d\theta }$ suppresses rapid changes in assistive force, and the stiffness term $K\cdot F_{\mathrm{int}}\left(\theta \right)$ maintains the system’s compliance, thereby balancing safety and assistive efficacy. This force feedback mechanism effectively prevents patient discomfort caused by excessive assistive forces while maintaining necessary assistance. This is of great significance in preventing potential harm to patients during the respiratory assistance process.

Next, to achieve Low-Level Controller, the planned force $F_{plan}$ was converted into the actuator’s desired pressure $P_{\sup}^{ref}$ based on Equation 1:

$$P_{\sup}^{ref}\left(\theta \right)=\frac{F_{plan}\left(\theta \right)}{\alpha }$$ (20)

The physical characteristics of the pneumatic actuation system are described by the Bernoulli equation:

$$Q_{\sup}=C_{f}A\sqrt{\frac{2∆P}{\rho }}$$ (21)

Where $Q_{\sup}$ is the supply flow rate, $C_{f}$ is the flow coefficient, A is the orifice area, ρ is the fluid density, ∆P is the pressure difference. The $Q_{\sup}$ of the proportional control valve is proportional to the control current I , expressed as:

$$Q_{\sup}=k_{1}\cdot I$$ (22)

Where $k_{1}$ is the current-to-flow conversion coefficient. By substituting Equation 20 into Equation 22, the relationship between the supply pressure $P_{\sup}$ and the control current I can be derived as:

$$P_{\sup}=P_{in}-{\left(\frac{I}{k_{1}C_{f}}\right)}^{2}$$ (23)

Where $P_{in}$ is the source pressure (constant). To achieve precise tracking control of the desired pressure $P_{\sup}$ , a PD control law with a feedforward compensation term was designed based on Equation 23:

$$I\left(\theta \right)=k_{1}C_{v}\sqrt{P_{\text{in}}‐k_{P}\left(P_{\sup}^{\text{ref}}\left(\theta \right)‐P_{\sup}\left(\theta \right)\right)‐k_{D}\left(\frac{dP_{\sup}^{\mathrm{ref}}\left(\theta \right)}{d\theta }‐\frac{dP_{\sup}\left(\theta \right)}{d\theta }\right)}$$ (24)

Notably, Equation 24 introduces a nonlinear feedforward control term, where the square root operation compensates for the physical characteristics of the pneumatic system’s flow-pressure nonlinearity (Equation 21), significantly enhancing open-loop dynamic response. By adjusting the proportional gain $k_{P}$ and derivative gain $k_{D}$ , the system achieves rapid tracking of the desired pressure while ensuring closed-loop stability.

To analyze the system stability, the state variables are defined as $x_{1}=P_{\sup}^{ref}\left(\theta \right)-P_{\sup}\left(\theta \right)$ , representing the supply pressure error, and $x_{2}=\frac{dP_{\sup}^{ref}\left(\theta \right)}{d\theta }-\frac{dP_{\sup}\left(\theta \right)}{d\theta }$ , representing the rate of change of the supply pressure error. The Lyapunov function is selected as:

$$V=\frac{1}{2}x_{1}^{2}+\frac{1}{2}x_{2}^{2}$$ (25)

Substituting the control law in Equation 24 into the error dynamics in Equation 25 gives the time derivative of the Lyapunov function in Equation 26:

$$\dot{V}=\left(1-k_{P}\right)x_{1}\;x_{1}-k_{D}\;{\left(x_{2}\right)}^{2}$$ (26)

For $k_{P}$ > 1 and $k_{D}$ > 0, $\dot{V}$ ≤ 0 is satisfied, proving asymptotic stability. Experimental optimization results in the parameters $k_{P}$ = 2.6, $k_{D}$ = 0.5, ensuring stable system performance. The control parameters are shown in Table 2.

In summary, the high-level controller is mainly responsible for generating the assistive force. In contrast, the low-level controller is primarily in charge of planning and tracking the assistive force. During this process, the dynamic planning mechanism of the low-level controller plays a crucial role, effectively ensuring the safety and comfort during the respiratory assistance process. Meanwhile, its tracking mechanism precisely controls the supply pressure to successfully achieve the target assistive effect.

## 3 Experimental validation of assistive efficacy

### 3.1 Randomized controlled trial design and respiratory function evaluation metrics

This study aimed to validate the effectiveness of the proposed human-robot interaction control strategy in improving respiratory function. The experimental platform used an Intel NUC high-performance computing unit. A Python script was developed for data acquisition and analysis, enabling synchronous collection and processing of respiratory flow data. The experimental design followed a Randomized Controlled Trial (RCT) approach.

As a preliminary feasibility study, the experiment was conducted on healthy subjects to evaluate the system’s basic functionality. Eight healthy volunteers (all male) were recruited as experimental subjects, with their basic characteristics summarized in Table 3. The subjects were aged 24–28 years, with heights ranging from 170.0 to 186.7 cm and body mass indices (BMI) within the normal range (18.5–24.9). The inclusion criteria were as follows: (1) no history of respiratory diseases, (2) no cardiovascular diseases, (3) no musculoskeletal disorders, and (4) no recent participation in other medical experiments. The study protocol was approved by the Ethics Committee of the State Key Laboratory of Robotics, and all subjects provided informed consent.

**TABLE 3:** Basic characteristics of subjects in respiratory assist experiments.

Subject ID	Gender	Age	Height (cm)	Weight (kg)
S1	Male	25	180.5	80.5
S2	Male	24	170.0	70.0
S3	Male	25	183.9	83.9
S4	Male	24	174.0	74.0
S5	Male	25	186.7	86.7
S6	Male	28	170.1	64.5
S7	Male	23	176.7	75.7
S8	Male	24	170.1	72.5

**FIGURE 6:**
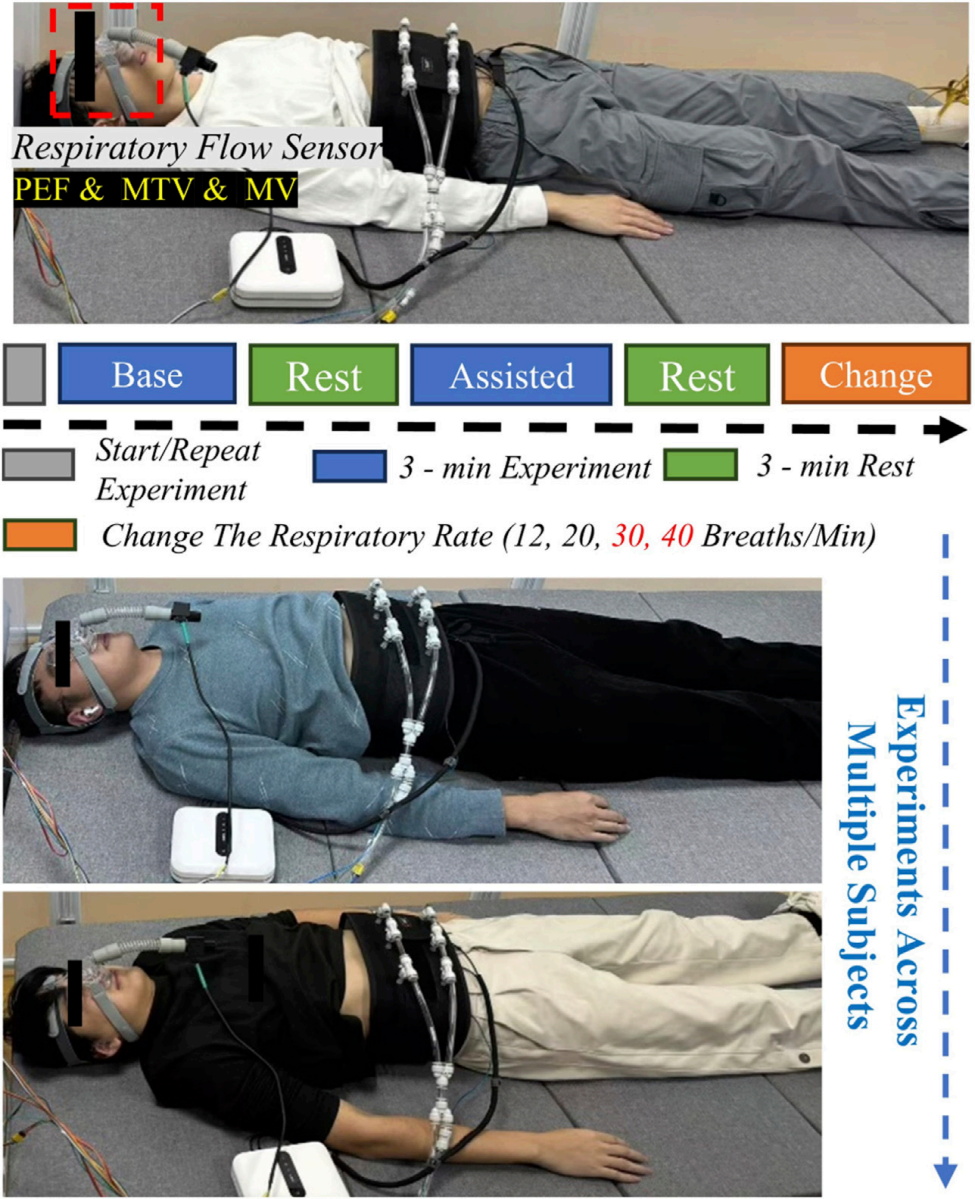
Schematic diagram of respiratory assistance experimental platform and process for healthy subjects.

The experiment was conducted under standardized supine conditions (Figure 6). Subjects wore the actuation unit, respiratory flow sensor, and were instructed to maintain a natural breathing pattern to ensure data reliability. A crossover experimental design was adopted, with each subject undergoing three sets of tests. Each set consisted of a baseline test under natural breathing and a robot-assisted test. Each test lasted 3 min, with a 3-min rest interval between tests to eliminate fatigue effects. During the experiment implementation, subjects first breathed naturally without the respiratory assistive robot, and their basic respiratory parameters were recorded as baseline data. Subsequently, guided by beeps, and considering the respiratory characteristics of normal individuals and ICU-AW patients, subjects were made to achieve respiratory frequencies of 12 breaths per minute (0.2 Hz), 20 breaths per minute (0.33 Hz), while 30 breaths per minute (0.5 Hz) and 40 breaths per minute (0.67 Hz) were set to simulate the breathing frequencies of ICU-AW patients with respiratory dysfunction respectively. Each frequency was tested three times. Upon completion, complete data from all eight subjects were collected (Figure 6).

The effectiveness of the assistive robot was evaluated by estimating and analyzing key respiratory parameters in experiments with healthy subjects, including Peak Expiratory Flow (PEF), Mean Tidal Volume (MTV), and Minute Ventilation (MV), to assess its impact on improving respiratory function in subjects. Statistical significance is denoted by asterisks: one (*) for *p* < 0.05, two (**) for *p* < 0.01, and three (***) for *p* < 0.001.

This study established a multidimensional indicator system to objectively evaluate the effects of respiratory assistance. In terms of ventilation function, Minute Ventilation (MV) were used to measure respiratory efficiency and overall ventilation effectiveness. Additionally, Peak Expiratory Flow (PEF) and Mean Tidal Volume (MTV) were included in the evaluation system, reflecting airway patency and breathing depth, respectively.

These assessment indicators hold significant meaning. Schmidt et al. validated the reliability of respiratory physiological parameters such as MV in evaluating the effects of ventilator assistance, showing that improvements in airflow velocity and ventilation are positively correlated with patient prognosis (*p* < 0.05) (Schmidt et al., 2010). Through comprehensive analysis of multiple indicators, this experiment was able to evaluate the functionality of the respiratory assistance system comprehensively.

### 3.2 Experimental results validate the effectiveness of the control system

During the experiment in Section 3.1, all state variables were synchronously sampled and recorded, all state variables were synchronously sampled and recorded.

**FIGURE 7:**
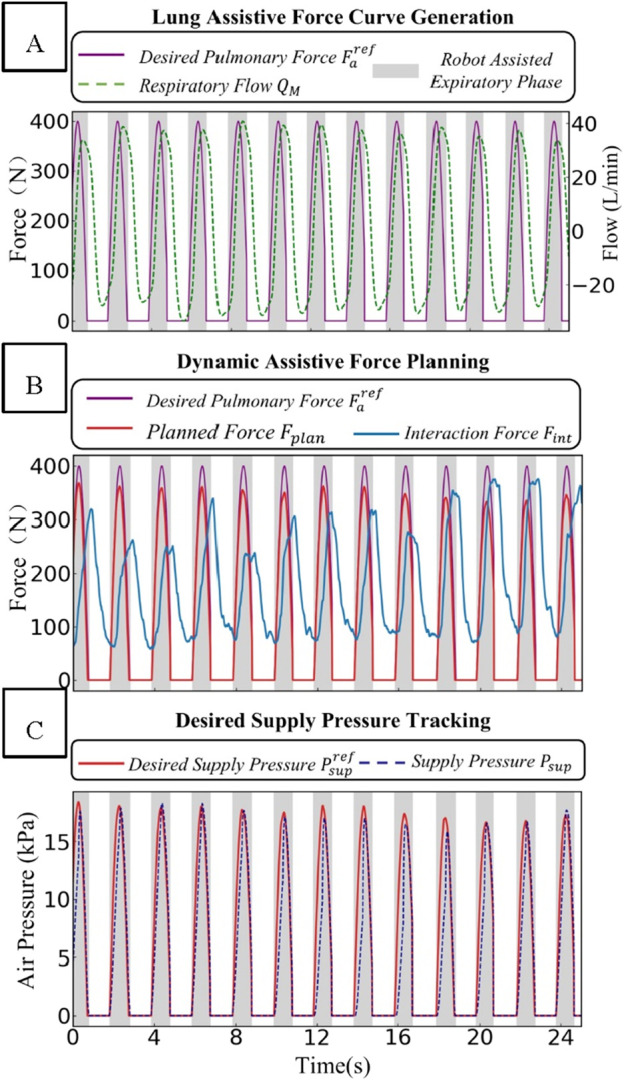
Schematic diagram of the dynamic changes of force and pressure-related parameters in the human-robot interaction control system. **(A)** Lung assistive force curve generation. **(B)** Dynamic assistive force planning. **(C)** Desired supply pressure tracking.

As shown in Figure 7, the experimental results fully validate the effectiveness of the control strategy. Figure 7 consists of three sub-figures, each representing a key component within the human-robot interaction control system. In the first component, as depicted in Figure 7A, the desired value of the lung assistive force is generated based on the respiratory flow and through the “Expected Pressure Curve Generation” module. The purple line in the figure represents the Desired Pulmonary Force $F_{a}^{ref}$ , the green dashed line represents the respiratory flow $Q_{M}$ , and the gray area represents the robot-assisted interval.

In the “Dynamic Assistive Force Planning” part, the rated force value is determined using the real-time feedback of the human-robot interaction force $F_{\mathrm{int}}$ . The compliance characteristics are derived from the terms $B\cdot \frac{dF_{\mathrm{int}}\left(\theta \right)}{d\theta }$ and $K\cdot F_{\mathrm{int}}\left(\theta \right)$ . As illustrated in Figure 7B, when the change rate of $F_{\mathrm{int}}$ is too high, the planned force $F_{plan}$ will decrease substantially, which helps ensure safety. Moreover, by modifying the parameters B and K , the elasticity of the actuator structure can be adjusted to offer personalized assistance to patients. Nevertheless, due to the pneumatic delay in sensors and pneumatic actuators, this elasticity adjustment mechanism has a time lag of 200 ms.

In the “Desired Supply Pressure Tracking” section, as depicted in Figure 7C, when the supply pressure $P_{\sup}$ of the actuator tracks the desired pressure $P_{\sup}^{ref}$ , the tracking error is −0.86 ± 1.7 kPa (mean ± standard deviation), and the response time is under 50 ms. This vividly illustrates the high-precision pressure-tracking performance of this section. Despite a certain error margin, the rapid response time enables the system to promptly adjust and get as close as possible to the desired pressure value.

In summary, the dual-layer control architecture coordinates force and pressure. Thanks to the rational design of the force-pressure conversion relationship, it can map the respiratory assistive force curve to actuator control and form a closed-loop control.

### 3.3 Quantitative evaluation of respiratory function and experimental validation

**FIGURE 8:**
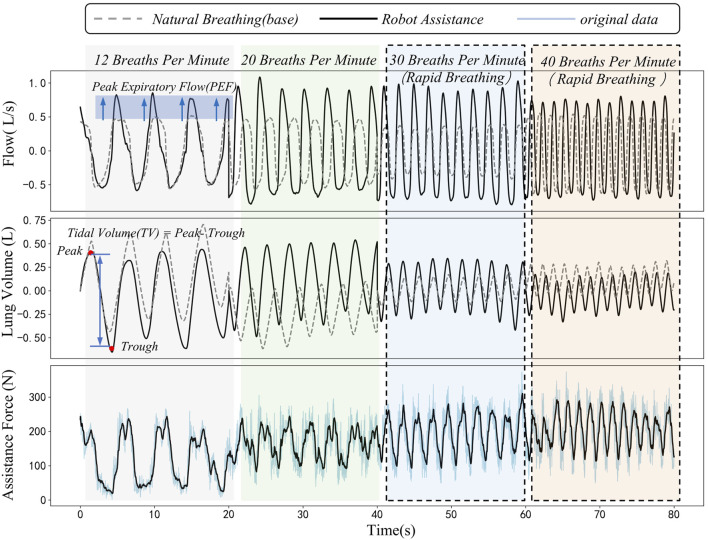
Dynamic comparison diagram of peak expiratory flow, tidal volume and assistance force during natural breathing and robot-assisted breathing at different respiratory frequencies.

This figure shows data on respiratory flow, tidal volume, and assistance force during natural and robot-assisted breathing at different frequencies (12, 20, 30, and 40 breaths per minute, with the respiratory frequencies of 30 and 40 breaths per minute simulating those of patients with impaired respiratory function). Figure 8 reveals that in the robot-assisted mode, across 12–40 breaths per minute, the Peak Expiratory Flow (PEF) has a significant increase compared to baseline. Tidal volume, calculated as the difference between end-expiratory and end-inspiratory lung volumes, is higher than baseline in nearly every cycle during robot assistance.

The third sub-figure displays the robot’s periodic assistance force. There are distinct differences in this force during human-robot interaction at different frequencies, likely due to dynamic changes in abdominal elastic parameters during breathing. The assistance force acts on the abdomen, synchronizing with the respiratory cycle to support respiratory muscles, thereby positively influencing respiratory function. During the experiment, the interaction force and its changes were closely monitored. The results indicated that the interaction force demonstrated stable periodic changes within the set safety limits. This suggests that the system is capable of not only effectively enhancing respiratory function but also ensuring the safety of subjects throughout the experimental process, thereby further validating the safety of the proposed method.

**FIGURE 9:**
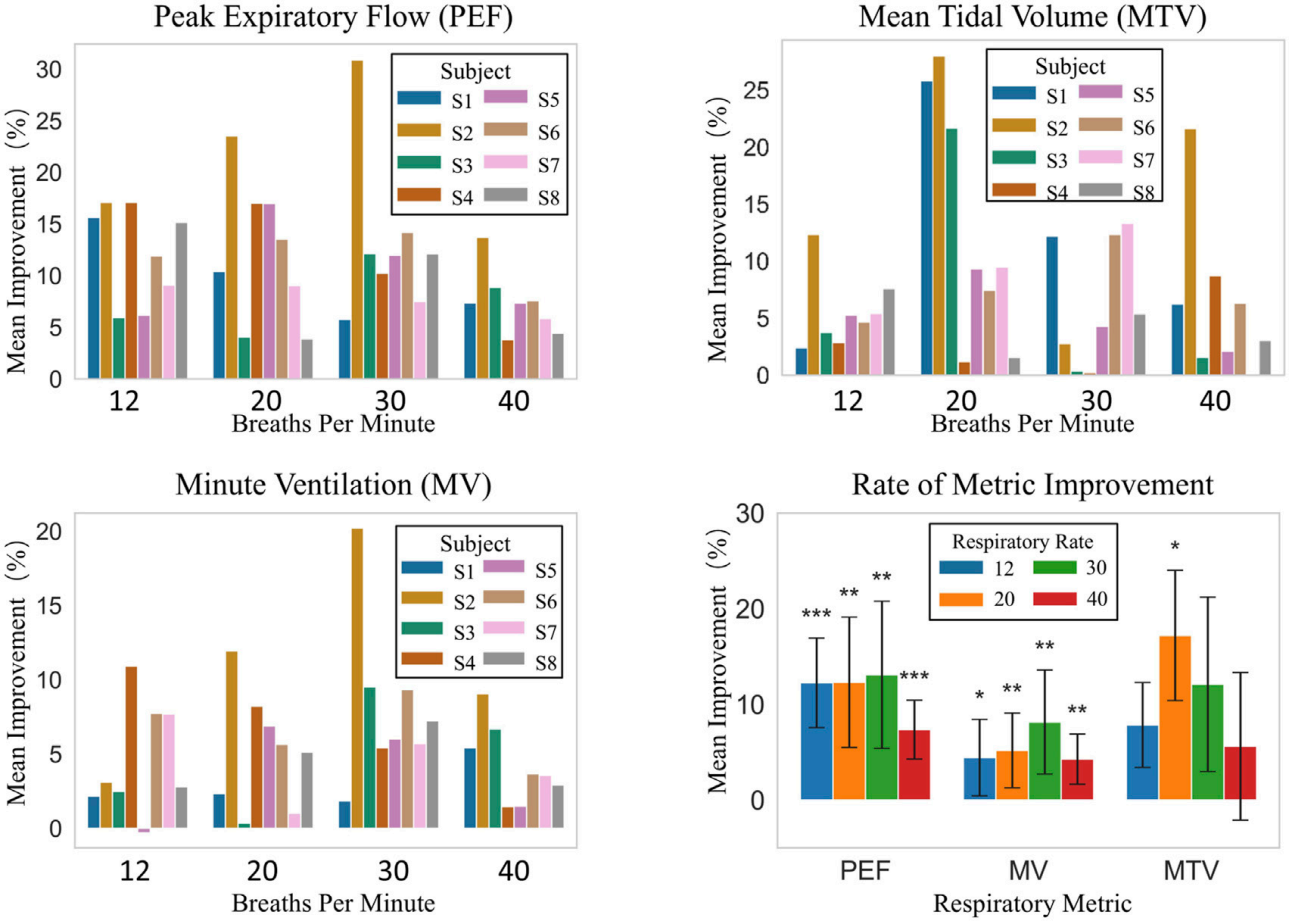
Respiratory metrics improvement: inter-subject comparison at different frequencies and overall analysis.

Figure 9 illustrates the average improvement of three parameters, namely, Peak Expiratory Flow (PEF), Mean Tidal Volume (MTV), and Minute Ventilation (MV), for each subject, along with their improvement scenarios at different respiratory frequencies (12, 20, 30, and 40 breaths per minute) under natural breathing (baseline) and robot-assisted breathing conditions.

As shown in Figure 9, improvements in PEF, MTV, and MV among individual subjects vary with respiratory frequencies. At 12 breaths/min, PEF improvement rates for S1–S8 range from 5.93% to 17.09%. At 20 breaths/min, S2 has a 23.52% improvement, while S8 only 3.88%. At 30 breaths/min, S2’s improvement is 30.87% compared to S3’s 12.11%. At 40 breaths/min, S2 improves by 13.70% and S4 by just 3.80%. MTV and MV show similar trends. For example, S7 has a large MTV increase across frequencies, while S1, S3, etc., have small increases. Overall, despite different improvement degrees, robot-assisted breathing enhances respiratory function for all subjects.

Overall statistical analysis, as presented in “Rate of Metric Improvement” in Figure 9 which shows the comparison with the baseline, indicates that robot-assisted breathing enhances respiratory-related indicators. The overall average improvement rates are 20.12% for PEF, 19.69% for MTV, and 5.46% for MV. At different respiratory frequencies: at 12 breaths/min, PEF improves by 12.25% with high statistical significance, and MV by 4.43% with statistical significance. At 20 breaths/min, PEF and MTV improve by 12.30% and 17.21% respectively, both reaching statistical significance. At 30 breaths/min, PEF improves by 13.09% and MV by 8.15%, with significant effects. At 40 breaths/min, PEF improves by 7.36% with high statistical significance.

Clearly, PEF and MV show high statistical significance at multiple frequencies, indicating a stable and significant effect of robot-assisted breathing on enhancing these two indicators. Although MTV’s overall significance is less than the other two, its 20 breaths/min improvement is also highly significant, suggesting robot-assisted breathing can improve MTV as well. In conclusion, robot-assisted breathing is effective in improving PEF, MV, MTV. In particular, for PEF and MV, high statistical significance is demonstrated at most respiratory frequencies.

**FIGURE 10:**
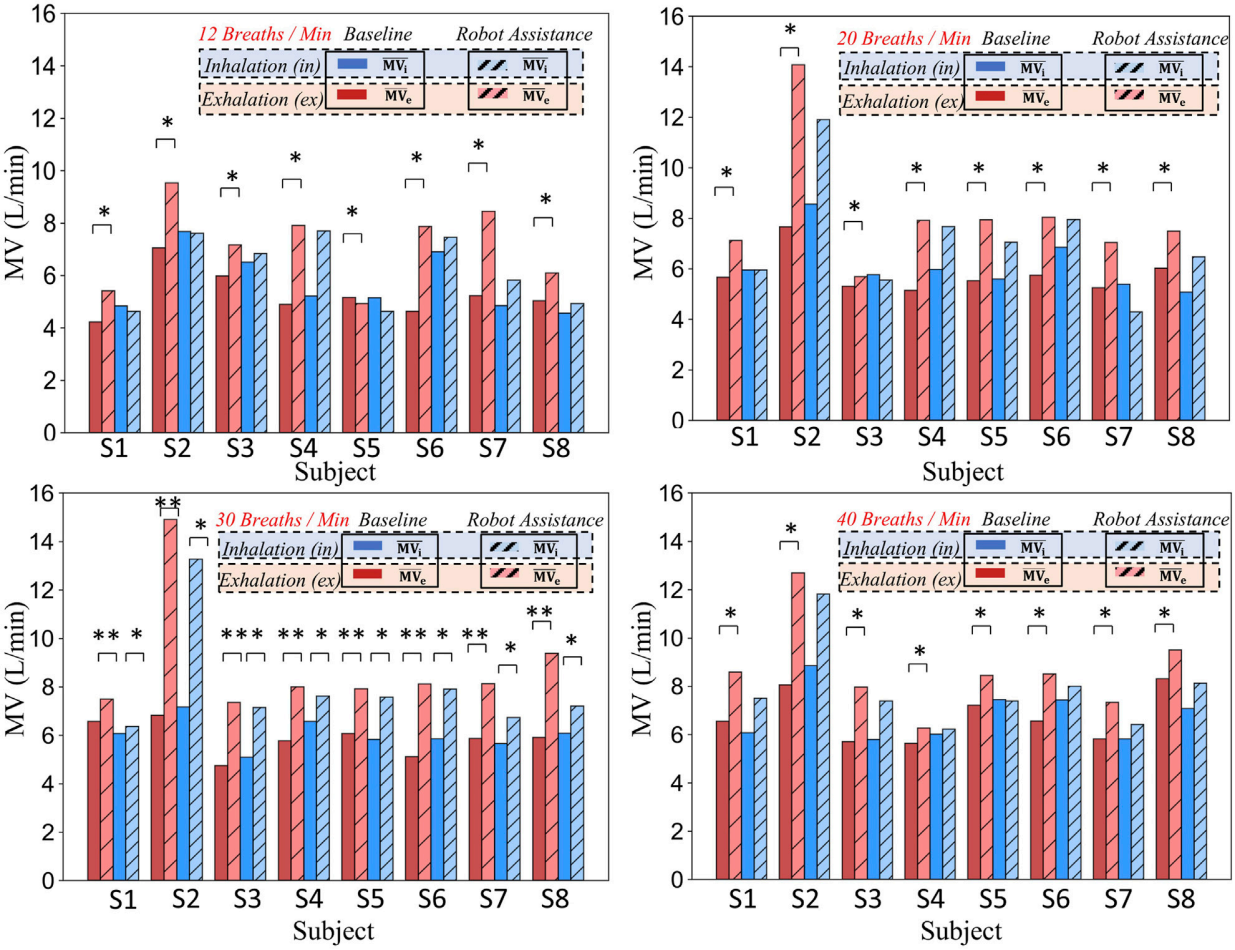
Comparison of minute ventilation in different respiratory phases (inhalation and exhalation) for eight subjects at various respiratory frequencies under baseline and robot-assistance conditions.

Figure 10 consists of four sub-graphs, which respectively illustrate the minute ventilation (MV) during the inspiratory and expiratory phases for eight subjects under natural breathing (baseline) and robot-assisted breathing conditions at four respiratory frequencies: 12 breaths per minute, 20 breaths per minute, 30 breaths per minute, and 40 breaths per minute. Different colors and patterns are employed to distinguish between the two conditions.

To illustrate that providing assistance solely during the expiratory phase can enhance the respiratory function during the inspiratory phase and consequently improve the overall respiratory function, the respiratory data were partitioned into two distinct phases: inspiration (i) and expiration (e), each clearly marked with a subscript. The inspiratory phase (i) encompasses the transition from the resting state to the apex of inspiration, effectively representing the active ventilatory capacity. Conversely, the expiratory phase (e) pertains to the passive expiratory process subsequent to inspiration, serving as an indicator of the efficiency of passive exhalation.

By meticulously calculating the data for these two phases under two scenarios-natural breathing and robot-assisted breathing-we are able to precisely quantify the impact of the robot on both inspiration and expiration. Dark-colored bars are employed to signify the baseline (natural breathing) data, while hatched light-colored bars represent the data obtained during robot-assisted breathing. This visual representation facilitates a comprehensive comparative analysis of the overall respiratory function, clearly demonstrating that expiratory-phase assistance can positively influence inspiratory function and lead to an overall enhancement of respiratory performance.

Taking specific data as an example, when the respiratory frequency is 20 breaths per minute, for subject S2, the expiratory ${\text{MV}}_{e}$ is 1.27 L/min and the inspiratory ${\text{MV}}_{i}$ is 1.42 L/min at baseline. After robot assistance, the expiratory ${\text{MV}}_{e}$ significantly increases to 2.34 L/min, and the inspiratory ${\text{MV}}_{i}$ rises to 1.98 L/min. As can be observed from the figure, the increase in inspiratory MV for subjects at this respiratory frequency is statistically significant. Moreover, at all tested respiratory frequencies, the increases in MV during both respiratory phases are statistically significant.

In conclusion, whether from the data of specific subjects or the overall presentation in the figure, the respiratory-assisting robot demonstrates a notable enhancement of the expiratory function for subjects. At specific respiratory frequencies, it also significantly improves the inspiratory function. Given that all these improvements are statistically significant, it indicates that the robot can effectively strengthen the expiratory function of subjects and, to a certain extent, enhance the inspiratory function, thereby improving the overall respiratory function.

## 4 Discussion

### 4.1 Advantages of the dual-layer control strategy

The system adopts a Dual-layer control strategy mainly considering the particularities of the application objects, such as patients with respiratory dysfunctions like ICU-AW patients. These patients have weak respiratory muscles, complex respiratory states, and are sensitive to external forces. For them, safety and adaptability during the respiratory assistance process are of utmost importance.

In terms of interactive force feedback, this study introduces a dynamic adjustment control method, enhancing both safety and comfort. While existing approaches (Lee et al., 2022; Rezaei et al., 2024) employ virtual impedance control parameters to introduce elasticity into the control strategy. In contrast, this study proposes a future direction that involves the integration of high-precision sensors and impedance control, enabling active adaptation to respiratory rhythms and thereby enhancing the precision and effectiveness of assistance (Zhou et al., 2023).

As shown in the experimental results in Figure 7, the interactive force $F_{\mathrm{int}}$ consistently remains below the desired assistive force $F_{a}^{ref}$ , with a dynamic response time and steady-state error within acceptable ranges. Additionally, the tracking error of the actuator supply pressure $P_{\sup}$ relative to the desired pressure $P_{\sup}^{ref}$ is maintained within a narrow margin, demonstrating high-precision pressure tracking performance. Compared to existing studies (Lee et al., 2022; Zhang et al., 2024a), this research further enhances system safety through strict safety constraints (e.g., supply pressure $P_{\sup}$ ≤20 kPa, interactive force $F_{\mathrm{int}}$ ≤400), thereby avoiding overload or unsafe operating risks. This interactive force feedback mechanism not only ensures the compliance of the assistive force but can also be adjusted for different assistive tasks, thus improving the applicability of the system.

### 4.2 Limitations analysis

The limitations of this study mainly involved two aspects: Firstly, there were limitations concerning the subjects. Although the experimental design of this study validated the preliminary feasibility and safety of the human-robot interaction control strategy among healthy subjects, certain limitations still existed, primarily related to the selection of subjects and sample characteristics. Specifically, the experiment recruited only eight healthy male subjects, which constituted a small and gender-homogeneous sample size that did not adequately reflect the individual differences across different genders, ages, and physiological states. This sample limitation may lead to biases when generalizing the experimental results to a broader population (such as females, the elderly, or patients with respiratory diseases).

Secondly, in the study of robot-assisted breathing systems, the simplification of physiological models, while providing a theoretical foundation for system design and control strategies, also introduced certain limitations. The pulmonary dynamics model reduced the respiratory system to a single first-order spring-damper system, neglecting the branching structure of the airways, alveolar heterogeneity, and the localized effects of gas exchange during respiration. This simplification may lead to issues with mismatched assistive forces in the control system. Additionally, the identification of model parameters (such as airway resistance R and lung elasticity E ) relied on data obtained under specific experimental conditions, which may not fully reflect the dynamic variations in different physiological states (such as pathological or exercise states).

To address these limitations, future research could focus on several improvement avenues: (1) Considering non-linear and dynamic characteristics: Building on the existing model by incorporating non-linear spring-damper components and viscoelastic material models to better simulate the dynamic responses of thoracic and abdominal tissues during respiration. (2) Clinical validation and optimization: Conducting large-scale clinical trials to verify the accuracy and robustness of the model, and continuously optimizing the model structure and parameters based on clinical feedback, to provide more reliable theoretical support for the practical application of the human-robot interaction system.

## 5 Conclusion

The human-robot interaction control system designed in this study demonstrated satisfactory control performance in experiments. In the Low-Level Controller pressure control, within the effective working range, the response time was less than 50 ms, with a steady-state error of −0.86 ± 1.7 kPa (mean ± standard deviation), under the safety constraints of a 20 kPa pressure upper limit and a 400 N assistive force threshold.

In the 12–40 breaths per minute effective respiratory rate range, significant improvements were seen in peak expiratory flow (PEF), mean tidal volume (MTV), and minute ventilation (MV). PEF had an overall average increase of 20.12%, MTV an overall average enhancement of 19.69%, and MV an overall average growth of 15.5%. Statistically, the improvements in PEF and MV across the entire effective range were highly significant, while MTV was significantly improved at a respiratory rate of 20 breaths per minute.

Respiratory-phase-segmented minute ventilation (MV) measurements showed that the respiratory assistive robot enhanced expiratory function and improved inspiratory function at certain rates within 12–40 breaths per minute. The system uses a dual - layer control architecture. This architecture combines force-pressure joint control and interactive force feedback mechanisms. As a result, the system can synchronize with human respiratory rhythms and perform dynamic tracking. Ultimately, it improves respiratory function.

For future work, on a theoretical level, models with non-linear and dynamic characteristics, combined with machine learning, can be introduced to enhance the system’s adaptability and robustness. In terms of application and clinical implementation, expanding the experimental scale with diverse subjects and collaborating with clinical rehabilitation departments for long-term follow-up studies are needed to validate the system’s universality and reliability.

Specifically, future research will focus on: (1) Integrating data-driven and mechanistic models to deepen the modeling of thoracic and abdominal dynamics and lung non-linearities for optimized control strategies; (2) Developing a more lightweight and high-precision hardware system to improve portability and practicality; (3) Conducting large-scale, diversified clinical trials to verify the system’s effectiveness and safety across different physiological states, providing a stronger basis for clinical applications.

## Data Availability

The original contributions presented in the study are included in the article/supplementary material, further inquiries can be directed to the corresponding authors.
